# Clocking the anisotropic lattice dynamics of multi-walled carbon nanotubes by four-dimensional ultrafast transmission electron microscopy

**DOI:** 10.1038/srep08404

**Published:** 2015-02-12

**Authors:** Gaolong Cao, Shuaishuai Sun, Zhongwen Li, Huanfang Tian, Huaixin Yang, Jianqi Li

**Affiliations:** 1 Beijing National Laboratory for Condensed Matter Physics, Institute of Physics, Chinese Academy of Sciences, Beijing 100190, China; 2 Collaborative Innovation Center of Quantum Matter, Beijing 100084, China

## Abstract

Recent advances in the four-dimensional ultrafast transmission electron microscope (4D-UTEM) with combined spatial and temporal resolutions have made it possible to directly visualize structural dynamics of materials at the atomic level. Herein, we report on our development on a 4D-UTEM which can be operated properly on either the photo-emission or the thermionic mode. We demonstrate its ability to obtain sequences of snapshots with high spatial and temporal resolutions in the study of lattice dynamics of the multi-walled carbon nanotubes (MWCNTs). This investigation provides an atomic level description of remarkable anisotropic lattice dynamics at the picosecond timescales. Moreover, our UTEM measurements clearly reveal that distinguishable lattice relaxations appear in intra-tubular sheets on an ultrafast timescale of a few picoseconds and after then an evident lattice expansion along the radial direction. These anisotropic behaviors in the MWCNTs are considered arising from the variety of chemical bonding, i.e. the weak van der Waals bonding between the tubular planes and the strong covalent sp^2^-hybridized bonds in the tubular sheets.

Transmission electron microscopy (TEM) and electron diffraction, due to their fine spatial resolution, have played a very important role in the microstructural investigations of advanced materials. The experimental 2D and 3D micrographs in general could provide static structures with a remarkably high-spatial resolution. In particular, recent developments on the aberration-corrected TEM allow us to obtain images with a resolution on the sub-angstrom scale for crystalline samples^1^,^2^. On the other hand, the frontier of TEM instrument developments in the past decade is increasingly focused on the in-situ and time-resolved experimentation as reviewed in previous literatures^3^. For instance, the developments of 4D-UTEM and relevant experimental techniques have been used broadly to study ultrafast structural dynamics in solids^4^,^5^,^6^, and these investigations indeed have resulted in unprecedented insights into the lattice dynamics of solids undergoing structural evolution and phase transitions. In particular, recent studies on 4D-UTEM with single-electron pockets, space charge effects and their associated pulse broadening mechanisms have made remarkable progresses, reaching the femtosecond regime for imaging with electrons. Furthermore, the associated instrument developments are at present very rapid, frequently bringing new challenges and solutions to the new physical regimes in which these instruments operate^6^,^7^,^8^,^9^,^10^. It is now possible to drive material processes with various combinations of heat, gaseous and focused lasers and to capture the evolution using either conventional video-rate acquisition or ultrafast pump-probe method^11^,^12^.

In this Article we report our recent works on developing a new 4D-UTEM and anisotropic lattice dynamics of MWCNTs investigated by this 4D-UTEM. Our study clearly reveals that the intra-sheet dynamic responses and inter-layer lattice relaxation of MWCNTs occur evidently in two different timescales. They are fundamentally in correlation with the specific tubular structure and chemical bonding behaviors, i.e. the strong covalent bond in tubular sheet and weak van der Waals inter-layer bonding along the radial direction. These results demonstrate that our UTEM can be used for the study of nanoscale energy transport, electron-phonon coupling and lattice relaxation in picosecond timescales.

## Results

### Set-up and characterization of UTEM

**Figure 1:**
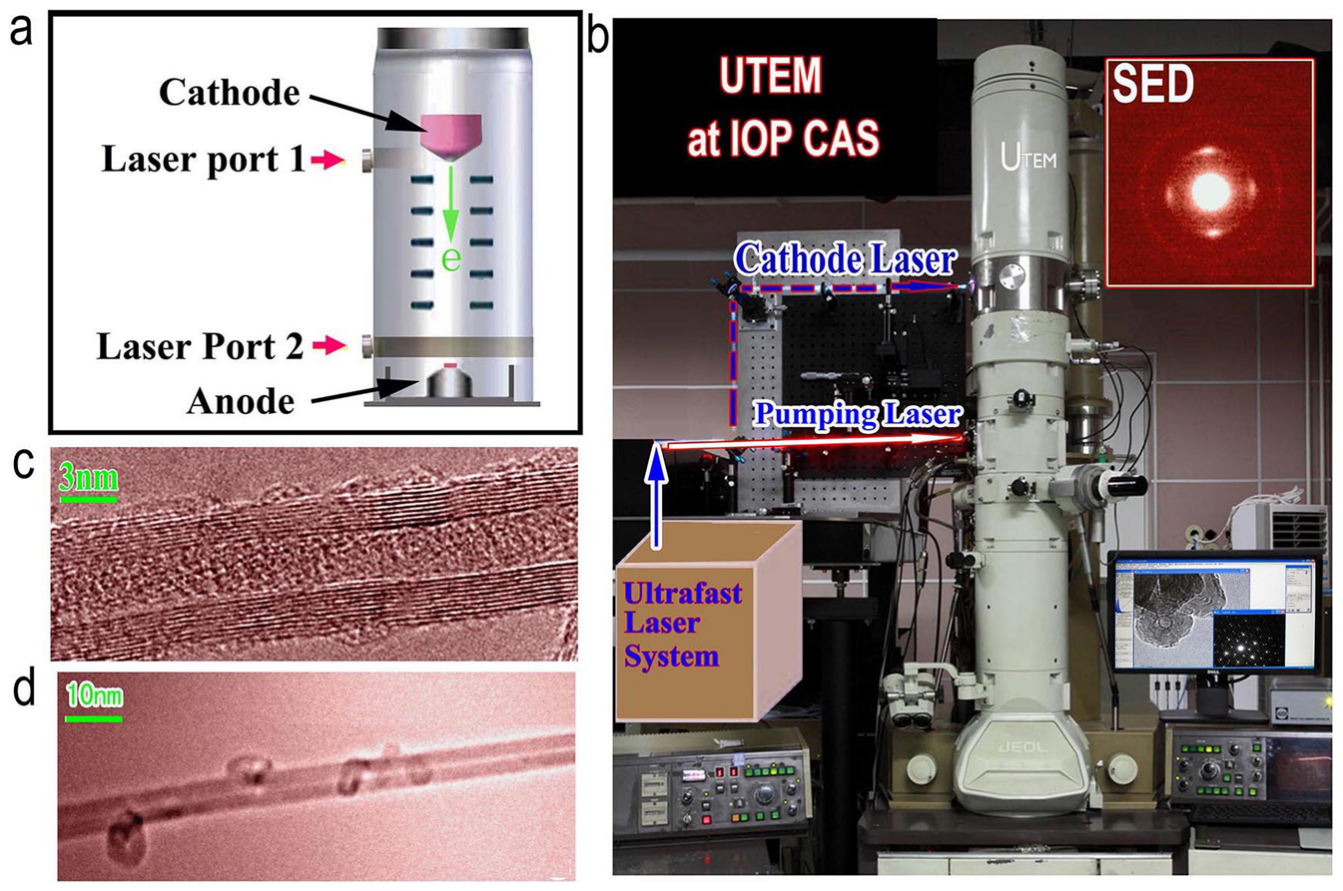
Photograph of 4D UTEM at IOP, CAS, together with the relevant experimental data. (a), A conceptual design for an UTEM gun in which the ultrafast laser can be introduced for driving photocathode from either laser port 1 or laser port 2. (b), A photograph of the UTEM at IOP, the inserted image is a single-electron diffraction (SED) pattern, demonstrating the ability of each individual electron to interfere with itself. (c) and (d), Micrographs for a MWCNT taken with thermionic and UTEM mode, respectively.

In past three years, we have focused our attention on modifications of the conventional TEM gun for development of an UTEM at Institute of Physics, Chinese Academy of Sciences (IOP, CAS). Previously, it is noted that research groups in LLNS (Lawrence Livermore National Laboratory)^13^ and Caltech (California Institute of Technology)^1^,^14^ have modified the TEM column structure by introducing an electron shift copper block between the anode and the condenser lens, moreover, a weak magnetic lens was also added in their UTEM to increase the number of electrons for time-resolved observations^13^. On the other hand, our project for developing UTEM started with modifications and improvements of an electron gun by re-designing the configuration, electronics and vacuum systems. It is expected that the modified gun can not only work well under photoelectron emission mode but also be operated as a conventional high-resolution TEM in a thermionic mode. Fig. 1a shows a schematic representation of our conceptual design for the UTEM gun, in which we can introduce the ultrafast laser for driving photocathode from either laser-port 1 or laser-port 2. Actually the laser-port 1 is designed to introduce photo-emission laser in a field-emission gun as similarly discussed in ref. 15. The laser-port 2 is designed for photo-emission associated with a thermionic gun. As a result of our three-year project and subsequent improvements, we have successfully developed a UTEM gun which works properly in a JEOL-2000EX for time-resolved imaging and conventional TEM observations as well.

Fig. 1b shows a photograph of the UTEM built at IOP, CAS. This microscope working with a modified electron gun can be operated for taking static and dynamic images as shown in the images Fig. 1c and d, respectively. The relevant technical details on developments of this UTEM are illustrated partially in the supplementary materials [Fig. S1–S2] and will be reported elsewhere. It is known that spatial resolution in time-resolved observations depends evidently on numerous factors, such as the sample instability associated with optical excitation and space charge broadening. Although we can see lattice of the graphitized carbon under ideal conditions as shown in Fig. S3^4^,^14^, in general we can obtain time-resolved images with a spatial resolution of about 0.5 nm as shown in Fig. 1c, this fact suggests that ultrafast electron microscopy can be a key technique to characterize the microstructural phenomena of nanoscale dynamics, such as phase segregations and the anisotropic lattice relaxations.

In order to operate our UTEM with a high-temporal resolution, the concept of single-electron imaging and relevant experimental techniques are critically important to eliminate the coulomb repulsion between electrons within a femtosecond pocket^4^. In fact, our recent investigations demonstrated that single electron diffraction and imaging are practically possible in the UTEM mode. The inserted image of Fig. 1b shows a single-electron diffraction (SED) pattern obtained from a MWCNT sample. This diffraction pattern is an accumulation of about 6 × 10^7^ single-electron pulses (with the integration time about 10-mins), the presence of clearly diffraction spots and rings in this pattern demonstrates the ability of each individual electron to interfere with itself, as similarly discussed in ref. 16. Moreover, it is also pointed out in previous literatures that as long as the number of electrons in each pulse is below the space-charge effect limit^4^,^17^, each pocket can actually have a few or tens of electrons, and the temporal resolution is still determined by the fs-optical pulse duration. Though precise measurement of the electron pulse durations under different operating conditions is a difficult work in the UTEM performance, we can efficiently estimate the duration of photo-electron pockets based on the UTEM parameters using the method as reported in ref. 17. e. g. when our UTEM is operated with the voltage of 160 kV, the electron numbers per pocket should be less than 200 electrons to achieve an optimum temporal resolution of 1 ps or less, as clearly illustrated in supplementary materials (Fig. S2 and Table S1).

### Ultrafast lattice dynamics of MWCNTs

**Figure 2:**
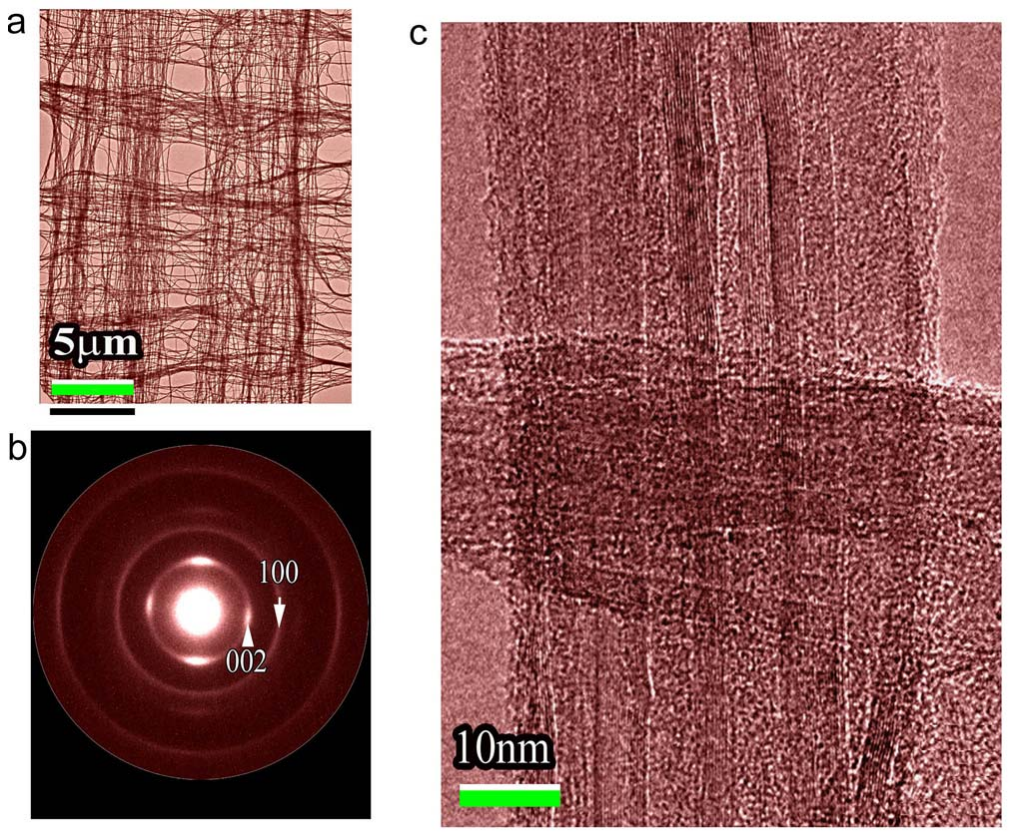
Microstructural properties of MWCNT samples for UTEM observations. (a), The textured MWCNTs on a TEM Cu-grid. (b), Electron diffraction pattern from the MWCNTs clearly show the (002), (004), (100) and (110) reflections. The decussating nantubes can yield a good signal-to-noise ratio in the ultrafast diffraction observations. (c), High-resolution TEM image shows lattice structure of well-aligned nanotubes.

We now turn to discuss the experimental results on the ultrafast lattice dynamics of MWCNTs. Because of the nearly one-dimensional electronic structure, electronic transport in metallic CNTs could occur ballistically over long lengths along the axial direction and phonons also propagate easily along the CNTs, the room temperature thermal conductivity for an individual CNT is reported to be greater than that of natural diamond and the basal plane of graphite^18^,^19^. Importantly, our UTEM observations in MWCNTs directly reveal the presence of remarkable anisotropic dynamic properties arising from electron-phonon coupling, phonon relaxation and lattice strain. The MWCNT samples used in present study were arranged in well-aligned bunches or decussating patterns on the conventional TEM Cu-grids as shown in Fig. 2. As a result, this kind of MWCNT samples with a thickness of 20 nm–30 nm is suitable for the fs-laser excitation during UTEM observations. In order to clearly visualize the structural dynamics in MWCNTs, an ultrafast fs laser system with the duration of 300 fs (wavelength of λ = 520 nm) was used to excite the charge-carriers in the nanotubes, then the excited electrons in the delocalized π bond undergo a fast relaxation and subsequently result in visible changes in the lattice structure. These atomic-scale dynamics are carefully analyzed by UTEM observations at different time delays. Our experimental measurements clearly demonstrate the presence of visibly anisotropic dynamic nature along the CNT radial direction and within the tubular sheets, respectively.

**Figure 3:**
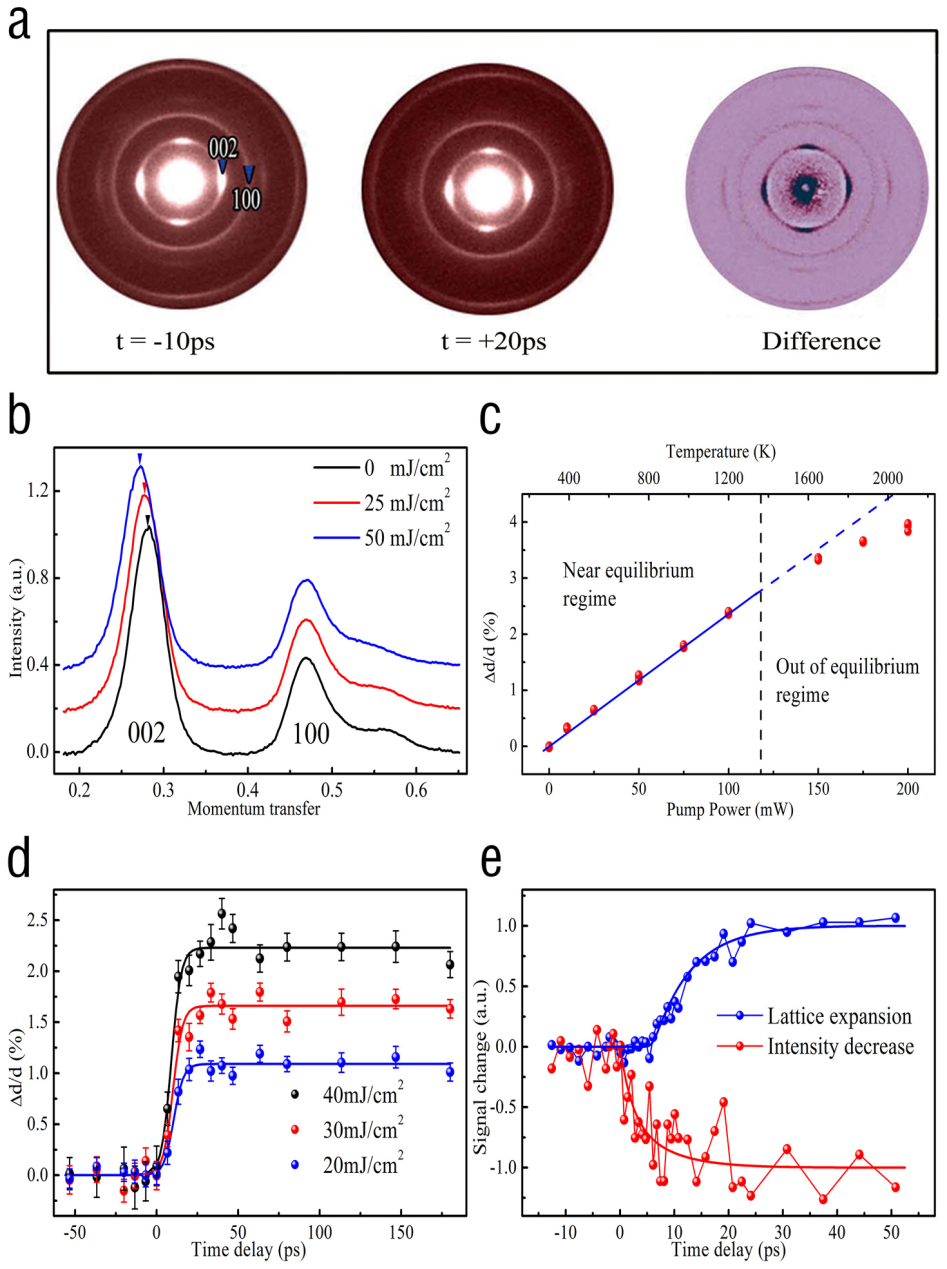
Time-resolved experimental data for lattice dynamics along the radial direction of the MWCNTs. (a), Temporal frames of diffraction patterns for negative time delay (t = −10 ps), positive time delay (t = 20 ps), and diffraction difference clearly illustrating the shift of the (002) reflection. (b), Radial integrated 1D diffraction curves for three different laser powers at the time delay of t = 20 ps, showing the progressive move of (002) peak with the increase of pumping power. (c), Fluence dependence of inter-planar expansion as measured at a time delay t = 20 ps, dynamic anomalies occur for heating laser power larger than 120 mW. (d), Temporal evolution of lattice expansions along the radial direction with laser fluences of 20 mJ/cm^2^, 30 mJ/cm^2^, 40 mJ/cm^2^, respectively. (e), The rate of diffraction change revealing the (002) intensity decay and inter-planar expansion with time, the diffraction intensity has been normalized to the negative data. The notable intensity decay appears at about 6 ps earlier than the notable lattice expansion.

One of the most notable structural features as revealed in UTEM observations is the appearance of visible lattice expansion along the tubular radial direction following the laser excitation. Fig. 3a shows two typical electron diffraction patterns obtained at the negative (−10 ps) and positive (20 ps) time delays for a fluence of 25 mJ/cm^2^. These diffraction patterns are taken from the textured MWCNTs with a decussating pattern (see Fig. 2), so it shows apparently the (002) diffraction spot (d = 3.4 Å) and (100) (d = 2.45 Å) diffraction ring that can be used to study temporal evolutions associated with lattice relaxations. For facilitating the comparison, diffraction difference between these negative and positive patterns are also displayed in the right frame of Fig. 3a, illustrating the visible shift for the (002) spots arising from thermal expansion along the radial direction. Careful analysis on the diffraction results obtained from a few well-characterized samples suggests that MWCNTs generally show visibly anisotropic dynamic responses in lattice spacing,i.e. the (002) and (004) peaks often shift their positions continuously with the increase of laser fluence, on the other hand, the (100) and (110) reflections show relatively small changes in their positions as also discussed in the following context. For the better view of these dynamic features, a few diffraction patterns were radial integrated to form a one-dimensional curve in which the position shifts for (002) spots can be clearly recognized. All data shown in Fig. 3b are taken at the time delay of 20 ps with laser fluences of F = 0, 25 mJ/cm^2^ and 50 mJ/cm^2^, respectively. Position changes for (002) diffractions are clearly indicated by a few arrows.

Fig. 3c shows radial expansion changes following with laser excitations at the positive time delay of 20 ps. It is demonstrated that when the sample is excited under moderate fluences of below 120 mW (~60 mJ/cm^2^), the interlayer expansion is proportional to the pump power and shows a linear increase in this regime. The maximum atomic motion, as initiated by ultrafast laser heating (~10^14^ K/s^−1^), is found to be around 0.10 angstroms along the radial direction. On the other hand, the radial expansion at higher values of fluence (e.g. larger than 150 mW) shows up a notable nonlinear tendency towards saturation, this fact suggests that high laser excitation could induce defect structures and even damage the atomic structure of MWCNTs, as similarly discussed for graphite in the far-from-equilibrium regime^20^ and also noted in our TEM examinations. In Fig. 3c we also display the calculated lattice temperatures based on our experimental data and thermal expansion coefficient of α_002_ = 2.6 × 10^−5^ K^−1^ 21, similar temperature rises can also be obtained from the pumping power and thermal parameters for MWCNTs.

Fig. 3d depicts the time dependence of the inter-planar space for three different laser powers corresponding to the fluence of 20 mJ/cm^2^, 30 mJ/cm^2^ and 40 mJ/cm^2^, respectively, where the heating laser beam diameter on the specimen is 60 μm. It is clearly recognizable that the thermal expansion along the radial direction increases evidently in MWCNTs with the increase of pumping fluences. The characteristic time for lattice expansion from a monoexpenential fit is 4 ± 1 ps, which is much faster than the reported data as measured using a laser duration of 16 ps^22^. Careful examinations reveals that this radial expansion occurs at the time delays between 6 ps to 18 ps, which could keep for hundreds of picoseconds depending on the thermal diffusion for the UTEM samples and then the recover to the pre-heating state. Moreover, one of the most critical issues concerned in present study is the correlation between this remarkable radial lattice expansion and the electron-phonon interaction in MWCNTs. We therefore have examined the diffraction intensity decays following with the laser excitation. It is commonly found that the diffraction intensity not only has a notable change accompanying with the inter-planar expansion but also shows up faster rate than the radial expansion as typically illustrated in Fig. 3e. Careful analysis suggests that this intensity decay results firstly from the electron–phonon coupling occurring chiefly within tubular sheets as discussed in flowing context.

### Anisotropic dynamic behaviors

It is noted that in our study the (002) peak often shows notable diffusive feature due to lattice defects and complicated microstructure of MWCNTs. Therefore the lattice responses in the intra-tubular plane have also been carefully measured in our UTEM observations. One of the most remarkable features as revealed in the following text is the presence two notable dynamic processes following the laser excitation: the ultrafast dynamic response primarily occurs within the tubular sheets with the time constant of 2.5 ± 1 ps associated with a small lattice expansion along the tubular axial direction and another one occurs relatively slower yielding visible atomic motions along the radial direction.

**Figure 4:**
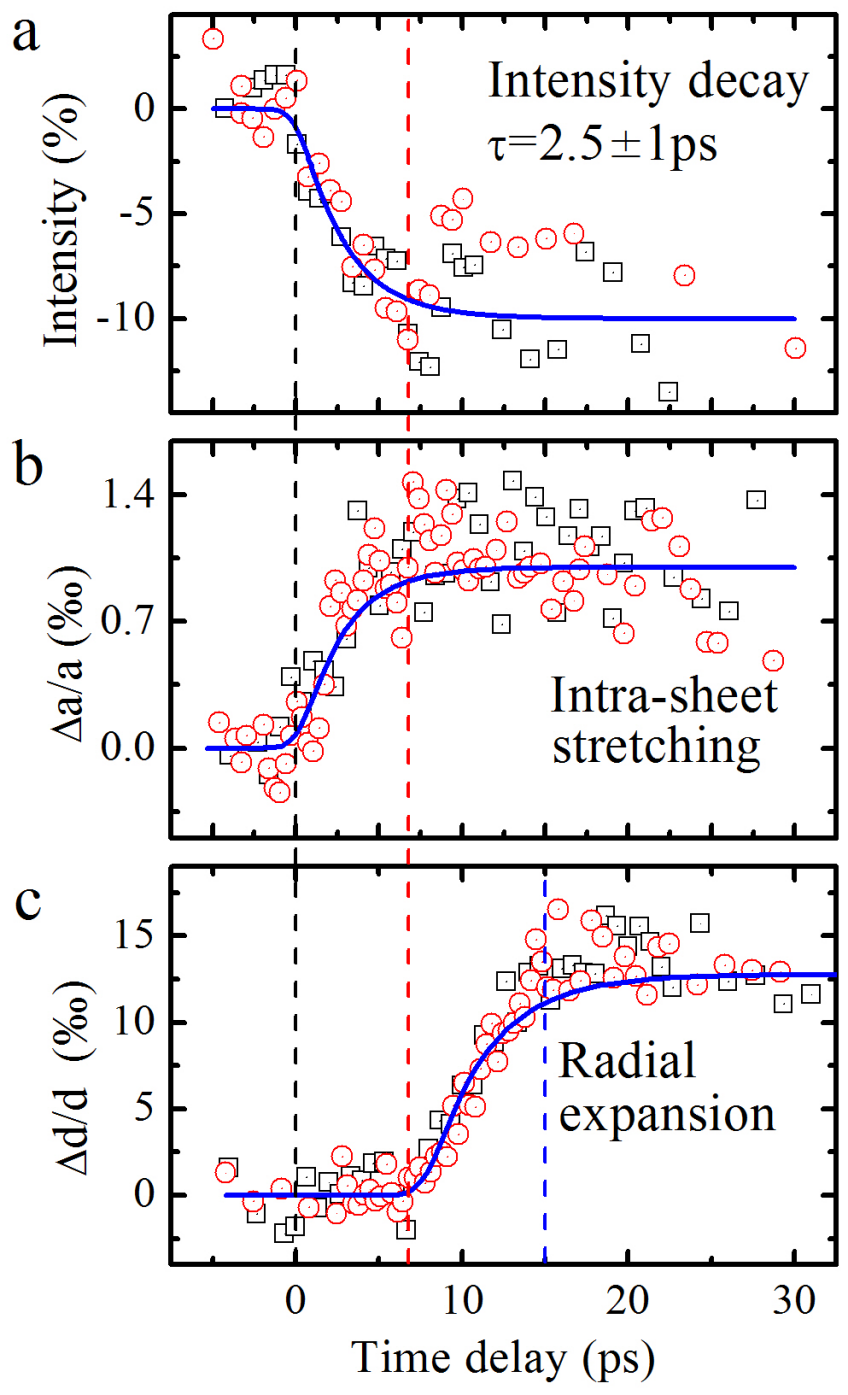
Temporal evolution of the diffraction signals on MWCNTs taken within the intra-sheet, the typical data on inter-planar expansion along the radial direction is also shown for comparison. (a), Ultrafast decay of the (100) diffraction intensity with a time constant of 2.5 ± 1 ps, suggesting the presence of a strong electron-phonon coupling in the tubular structures. (b) and (c), Comparison of the time dependence of lattice expansions as observed within the tubular sheets and along radial direction, demonstrating the anisotropic lattice relaxations in two different timescales. All of the three curves were fitted by the exponential decay function convoluted with the instrument response function, while the third curve had a time delay of 6 ps relative the time zero. The instrument response function is set as a Gaussian function with a FWHM of 1 ps.

In Fig. 4a, we plot the measured diffraction intensity for (100) ring as a function of the delay time, illustrating the structural change in the tubular sheets. The shown data were normalized to the average intensity obtained at negative times. After correcting for power losses, the pumping laser fluence is estimated to be around 30 mJ/cm^2^, it is found that the diffraction intensity is suppressed by 10% on the timescale of a few picoseconds as also demonstrate in another well-characterized sample. The atomic motion in correlation with the present phonon relaxation can be quantified by considering a time-dependent Debye-Waller factor. Now the vibration amplitude is time dependent and the diffraction intensity can be expressed as I(t)/I_0_ = exp(−s^2^δu^2^(t)/3), where I(t) is the intensity of a diffraction peak at a given time t after excitation, I_0_ is the intensity before excitation, s is the scattering vector, and δu^2^(t) is the mean-square atomic displacement. Because (100) is proportional to the square of the atomic displacements, the intensity suppression of 10% corresponds roughly to 1.6% change in atomic displacements (0.03Å). Furthermore, it is known that the rate of diffraction change is essentially in correlation with the electron-phonon coupling in the tubular sheets. We therefore have analyzed our UTEM data in comparison with results of the charge relaxation as measured in ultrafast optical spectroscopy^23^,^24^,^25^. Our experimental data reveals a time constant of 2.5 ± 1 ps for phonon response, which is in good agreement with the charge relaxation time observed in refs. 23, 24, 25.

Another noteworthy feature of the data shown in Fig. 4, and also partially discussed in Fig. 3e, is the apparently different nature for ultrafast responses observed in the intra-tubular sheets and along the radial direction. Though the thermal-expansion coefficient in the intra-sheets for MWCNTs is very small: α_100_ = (0 ± 0.1) × 10^−6^ K^−1^ at room temperature and α_100_ = 2.5 × 10^−6^ K^−1^ at high temperatures^21^, as a result, our UTEM measurements indeed can reveal this small intra-sheet expansion for laser fluence lager than 30 mJ/cm^2^. Fig. 4 shows temporal evolution of diffraction data obtained from a few well-characterized samples, it is recognizable that the first ultrafast response occurs between 0 and 6 ps, as indicated by diffraction intensity decay (Fig. 4a) and a small lattice expansion of about ~1‰ along the tubular axis direction (Fig. 4b), nevertheless the remarkable inter-planar expansion of about 1.5% as measured from the (002) spot begins at the time delay of about 6 ps (Fig. 4c, solid vertical line), these facts directly demonstrate that the lattice dynamics contain two anisotropic transient processes. In MWCNTs, because photoexcited carriers are actually anisotropically and preferentially coupled to specific phonon modes, so the three-temperature picture and relevant theoretical model should be invoked to understand the nature of the laser induced heating of electrons and phonons^26^,^27^. Importantly, according to the rate of diffraction changes and time scales for the specific electron-phonon coupling and anharmonic phonon-phonon interaction, our observations of ultrafast response in tubular sheets demonstrate the presence of a strong electron-phonon coupling and rapid lattice relaxations along the tubular-axial direction, which yields the fast decay of the diffraction intensity accompanying with phonon excitations. These remarkable structural features should be critical for understanding of specific electronic/thermal properties for this kind of one-dimensional structures.

## Discussion

**Figure 5:**
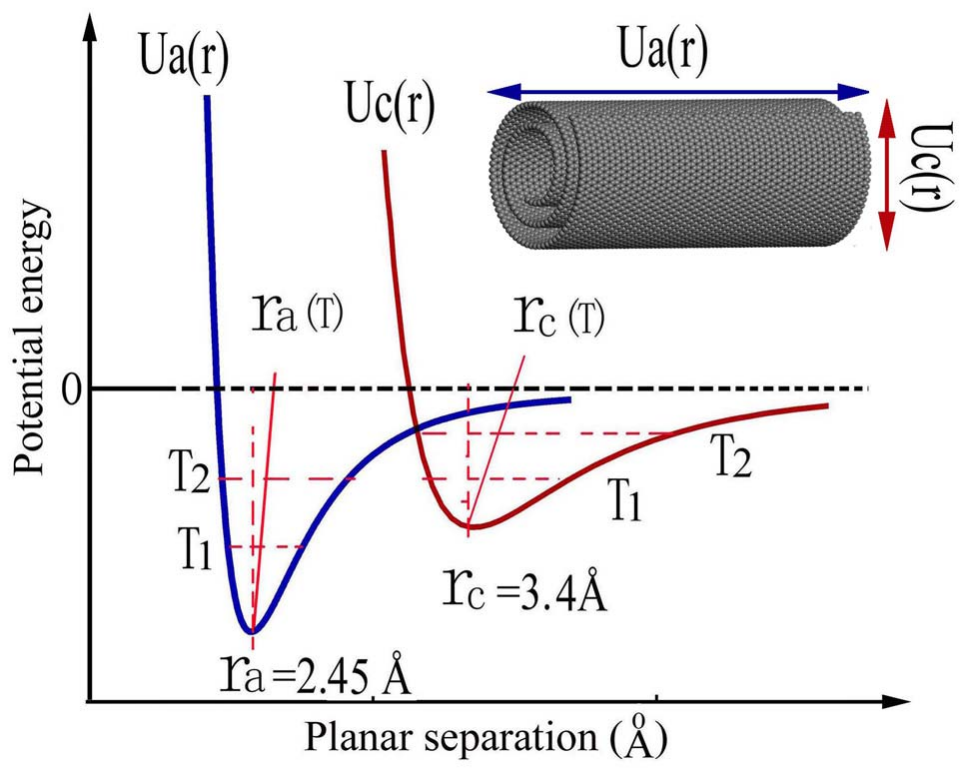
Schematic patterns for potential functions of the strong covalent bond in tubular sheet (U_a_(r)) and weak van der Waals inter-sheet bond (U_c_(r)) along the radial direction. At the room temperature we have r_a_ = 2.45 Å and r_c_ = 3.4 Å, it is clearly illustrated that r_c_(T) could increase visibly resulting from the laser heating owing to the presence of apparent anharmonic feature in U_c_(r), This weak inter-layered interaction can be also written in the Lennard-Jones form, U_c_(r) = 4ε[((σ/r)^12^ − (σ/r)^6^] with ε = 0.003 eV and σ = 0.34 nm as used for graphite. Inserted image shows the anisotropic structural features and related potential energies for a MWCNT.

The anisotropic dynamic feature are fundamentally in correlation with the variety of bonding types for MWCNTs. i.e. the weak van der Waals bonding between the annular layers and the strong covalent sp^2^-hybridized bonds in each sheet. It is known that the thermal expansion in solid results essentially from the anharmonic oscillations of atoms. In present case, MWCNTs contain the weak van der Waals bond between tubular planes and strong covalent bonds in tubular sheets. It is known that the covalent bonds often yield a quick dynamic response and a relatively small lattice expansion. In general, the potential energy for carbon bonding can be written as U = U(r) + αδr^2^ + βδr^3^ +…, in which the anharmonic terms could be visibly larger for van de Waals bond than that for the covalent bond^28^, Fig. 5 shows an schematic illustration on potential curves for both intra-tubular and inter-sheet interactions in the MWCNTs, in particular the asymmetric potential for the weak van der Waals bond is evidently exhibited. The average inter-sheet separation r_c_(T) could have a remarkable expansion following laser heating owing to the photo-excitation of anharmonic oscillation^28^,^29^. In the inserted image the anisotropic structural features and related potential energies are exhibited for a MWCNT. Interestingly, it is noted that the large interlayer thermal-expansion as observed for MWCNTs were comparable to those of the graphite interlayer spacing similar with what observed in the x-rays diffraction^21^, and this fact suggests that the MWCNTs are mostly adopted the scroll or mixed structures as illustrated in refs. 21 and 22.

Actually, the anisotropic behavior of the lattice dynamics in MWCNTs is essentially in correlation with the directional electron-phonon coupling. It is well known that the initial rate of electron-phonon scattering can be obtained through the equation^30^, 1/T_el-ph_ = 3hλ<ω^2^>/πk_B_Te, where T_el-ph_ is the characteristic coupling time constant and *ω* is the angular frequency of the coupled modes, k_B_ is the Boltzmann constant, and *λ* is a dimensionless parameter for effectiveness of the energy transfer between electron and hot-phonons. The angular frequency (*ω*) depends evidently on the strength of chemical bonds along a specific direction, so the weak van der Waals bonding between the tubular sheets results in a much lower angular frequency and a longer characteristic time along the radial direction as observed in our experiments. On the other hand, it is also noted that the MWCNTs often show up very similar structural features with graphite as demonstrated by the measurements of time-resolved optical spectroscopy^31^,^32^ and thermal expansions of inter/intra-planar lattice. Based on the UTEM measurements, our analysis clearly suggests that certain dynamic behaviors of MWCNTs are fundamentally comparable with what observed in graphite in which the typical electron-phonon coupling have been extensively studied by using ultrafast electron diffraction. For instance, it is well demonstrated that the strongly coupled optical phonons decay involves a complex interaction, a notable process decays to mid-frequency phonons with a time constant ranging from 700 fs ~ 2 ps have been observed^33^,^34^, and the excited state relaxes through further phonon-phonon pathways on the 6 ps–12 ps time scale^35^.

In conclusion, Our UTEM study clearly reveals a strong anisotropic nature of lattice dynamics for the MWCNTs, which are fundamentally in correlation with the specific tubular structure and chemical bonding behaviors, i.e. the strong covalent bond in tubular sheet and weak van der Waals inter-layer bonding along the radial direction. Importantly, our time-resolved structural analyses demonstrate that the intra-sheet dynamic responses and inter-layer lattice relaxation occur evidently in two different timescales. The intra-sheet dynamic feature is fundamentally in correlation with electron/phonon coupling and lattice relaxation along the tube-axial direction. These remarkable dynamic features should be critically important for understanding of the nanoscale energy conversion and phonon (electron) transport in this kind of one-dimensional tubular structures. With the development of UTEM, we can now study structural dynamics with high temporal and spatial resolution of advanced materials, we also plan to develop the aberration corrected UTEM equipped with STEM and EELS systems in next generation UTEMs, which can offer distinguished capability for probing the ultrafast structural dynamics in designed nanoscale materials and biological systems.

## Methods

The MWCNT samples used in present study were prepared using a method described by Huang et al.^36^, then the MWCNT samples for UTEM observations were fabricated in bunches and decussating patterns on the TEM Cu-grids as typically shown in Fig. 2a. Our high-resolution TEM investigations also demonstrated that the MWCNTs in each bunch are mostly well-aligned as clearly illustrating in Fig. 2b and Fig. 2c. These MWCNT films often have a thickness of 20 nm to 30 nm which is appropriate for the fs-laser excitation and UTEM studies. Moreover, the decussating pattern could yield clear diffraction spots with a fine signal-to-noise ratio which is essentially needed for analysis of ultrafast structural dynamics.

The UTEM built at our laboratory consists primarily of the femtosecond laser systems, UTEM gun, sample positioning chamber with the pumping laser-port, and CCD camera. The most significant new developments in this new machine are the modified UTEM gun which is capable for either time-resolved imaging or conventional high resolution TEM observations. There are two femtosecond laser systems with pulse durations of 100 fs (80 MHz) and 300 fs (1-1 MHz) are equipped with our UTEM for structural dynamic investigations. The time separation between laser pulses can be varied to allow complete heat dissipation in the examined specimen. We used the second femtosecond laser system for the ultrafast experiments of MWCNTs, which has relatively larger pulse energy and lower repetition rate. The output laser was split into two parts, one for pump laser after second harmonic generation (520 nm) and the other for probe laser after third harmonic generation (347 nm). The pump laser was focused to 60 μm by a convex lens with f = 500 mm and can obtain large pump fluence. The probe laser was focused to 100 μm to meet the size of cathode.

Experimental measurements for MWCNTs are preformed based on the laser pump-electron probe method. Firstly, the MWCNT specimens were excited by the pump laser introduced into the TEM column from a fused silica laser window, which can generate a series of successive dynamic process for ultrafast observations. Another pulsed probe laser was introduced into TEM gun for producing electron pulses which were subsequently accelerated in the high voltage TEM gun (80 kV–200 kV) and then scattered by the excited MWCNT samples, as a results, the time-resolved electron diffraction pattern and transmission images can be directly obtained for addressing the structural dynamics in examined area. In our UTEM experiments, the selected area apertures with the diameter of 5 μm–10 μm were often used to get diffraction data from a homogeneous region in the MWCNTs.

## Author Contributions

J.L., X.Y. and F.T. conceived the project, designed and modified the UTEM electron gun. S.S., Z.L. and G.C. established the ultrafast laser system. G.C., F.T. and J.L. installed the UTEM electron gun and commissioned the UTEM system. Z.L., F.T. and X.Y. prepared the sample of MWCNTs. J.L., S.S. and G.C. designed the ultrafast experiments and G.C., S.S. and Z.L. performed the experiments. S.S. and Z.L. analyzed the data. J.L. supervised the whole project. All authors contributed to the discussion of the results and to writing the manuscript.

## Supplementary Material

Supplementary Information Supplementary information
