# Supplementary material for: Clocking the anisotropic lattice dynamics of multi-walled carbon nanotubes by four-dimensional ultrafast transmission electron microscopy
Source: Sci Rep. 2015 Feb 12;5:8404. doi: 10.1038/srep08404 (PMC4325328; doi:10.1038/srep08404)
Supplement: Supplementary Information [file srep08404-s1.doc]

Clocking the anisotropic lattice dynamics of multi-walled carbon nanotubes by four-dimensional ultrafast transmission electron microscopy

Gaolong Cao^†^, Shuaishuai Sun^†^, Zhongwen Li, Huanfang Tian, HuaixinYang, and Jianqi Li^★^

# Supplementary information

Our development project for an ultrafast transmission electron microscope (UTEM) started with modifications of the configuration, electronics and vacuum systems based on a 200kV conventional TEM electron gun. It is expected that the modified gun can not only well work under photoelectronic emission mode but also be operated as a conventional high-resolution TEM in a thermionic (or a field emission) mode. In Fig.1, we show a photograph of the UTEM at IOP. Presently, it works properly for time-resolved imaging and conventional TEM observations as well.

**1. Cathodes for the UTEM gun**

During the developments of UTEM, it is commonly noted that the fundamental properties of the photo-cathodes play critical role for obtaining high-quality time-resolved images, so primarily we need good cathode sources for the UTEM gun assembly. Though we have measured and analyzed the experimental data obtained from a few good candidates in past years (e.g. LaB_6_, Ta, CeB_6_, SrB_6_), LaB_6_ is the only thermionic source commonly used in conventional high-resolution TEM observations, therefore, in this paper we will main report our experimental data on the implementation of photoelectric emission TEM gun on a JEOL-2000EX microscope with the LaB_6_ cathode plates.

**2. Time-zero determination**

It is known that there are a few fundamental issues have to be addressed for time-resolved UTEM observations, such as, the probe-pump synchronization, the space charge broadening, the source brightness and the electron coherence.

In order to perform pump-probe measurements on the UTEM mode, we firstly have adjusted our laser/TEM system and calibrate the synchronization between pumping fs laser and electron-pulse probe within subpicosecond precision. Actually, there are a few efficient methods that could well synchronize fs laser and electron pockets as used for UED and UEM systems. In our experiments, we found that it is convenient to use the untrashort electron pulses to image electron cloud of the surface plasma emitted from a metal surface following ultrafast laser illumination. Similar method is extensively discussed for imaging the electron shadow on a Cu TEM grid following fs-laser excitation ^1^. Moreover, this kind of measurements is also appreciated for the discussion of space charge effects and time resolution under specific conditions, Fig.S1 shows the time resolved experimental data to determine the position of t = 0.


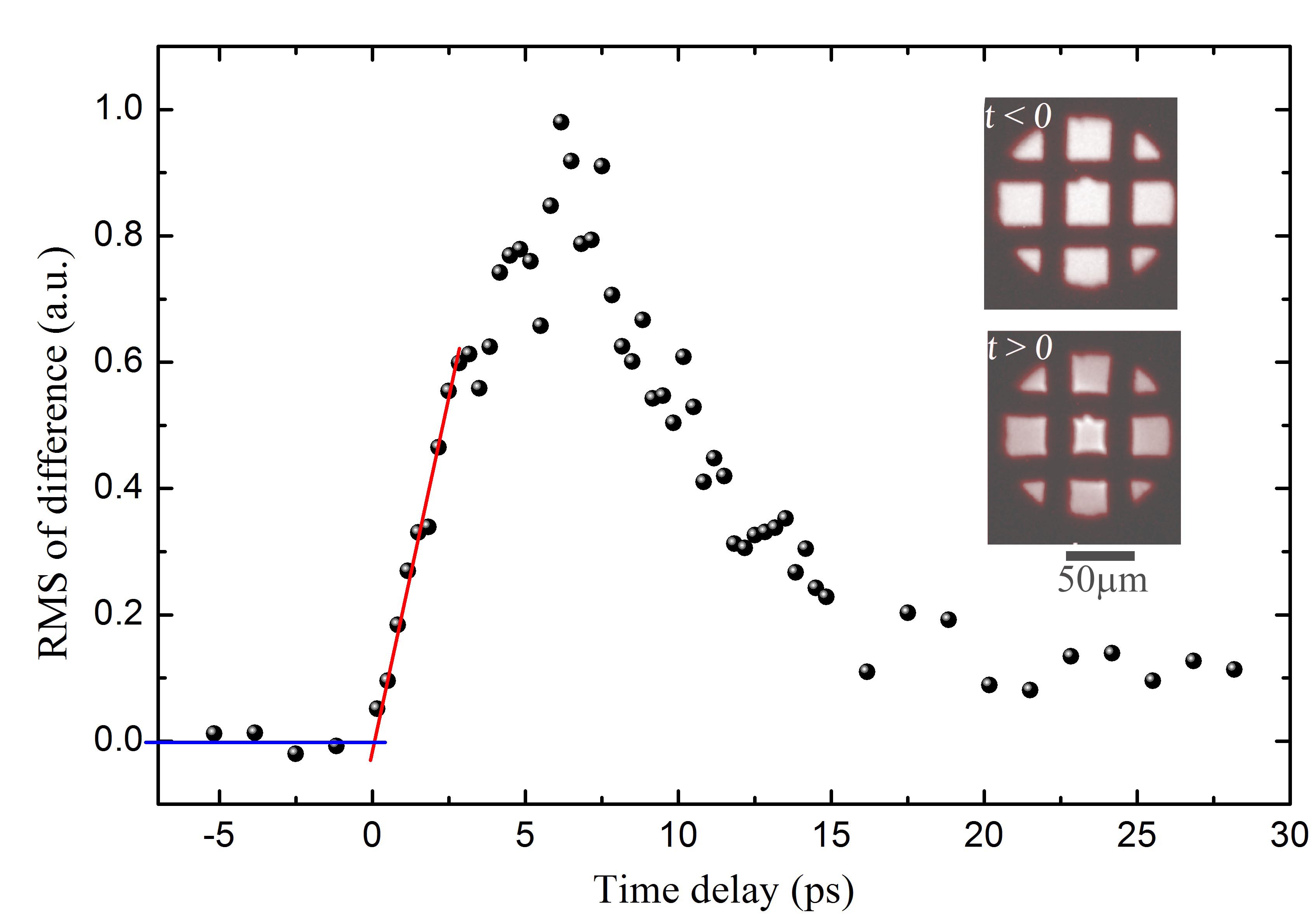


**Figure S1** **Temporal evolution of electron image for a Cu-grid excited by the fs laser pulses (λ = 520nm, F = 100mJ/cm^2^).** Normalized changes as measured from the root mean square (RMS) for signal difference as a function of the time delay. The copper TEM grid is excited by the pump laser at the center area to generate plasma cloud which pushes the probe electron pulses away from the Cu grid following photo-excitation. Time zero can be determined at the onset of plasma cloud formation. Inserted images shows typically the time resolved Cu grid images (400 mesh) before and after the arrival of pumping laser, in which the evident effects of excited surface plasma on the probe electron pulses can clearly recognized at the center region of the image.

**3. Space charge broadening effects**

It is known that temporal resolution of UTEM images are essentially limited by space charge effects, and certain ultrashort processes cannot be observed. In order to study the space charge broadening effects and duration of the photo electron pulse resulting from the alteration of cathode laser, we have performed our measurements for the probe laser between 10μJ/cm^2^ and 10mJ/cm^2^. Indeed we can see visible changes of the plasma cloud images, careful analysis suggests that the probe electron pockets are strongly broaden by space charge effects for the laser fluences larger than F=1mJ/cm^2^. In this project, we have used two femtosecond laser systems with the pulse durations of 100fs (λ=355nm, repetition rate 80MHz) and 300fs (λ=347nm, repetition rate 1MHz), respectively, they both can work properly for producing probe electron pockets.

**
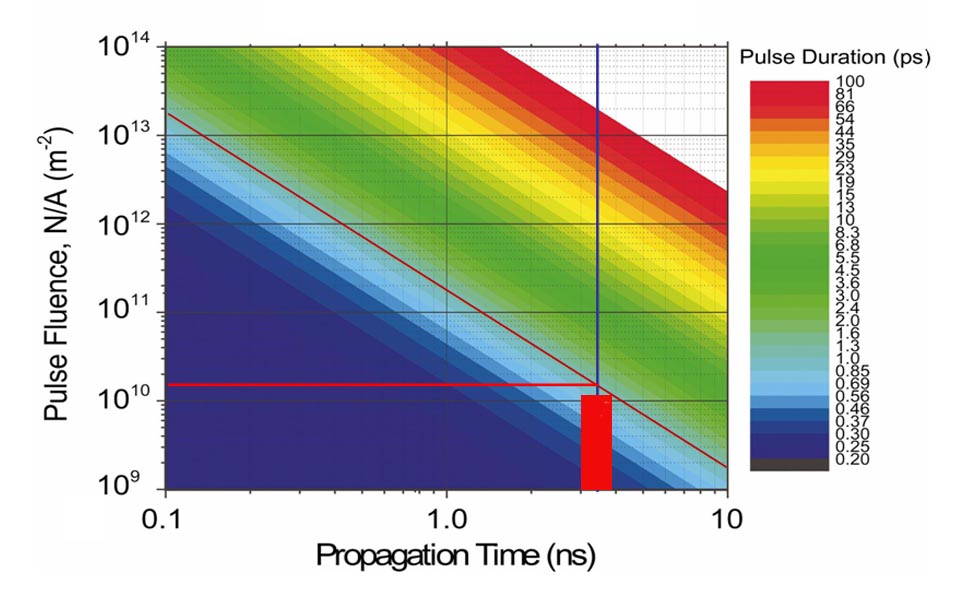
**

**Figure S2 Temporal broadening arising from space charge effects for femtosecond electron pulses**. This map illustrates clearly about pulse broadening effects for a given electron number density^2^. Our UTEM time-resolved experiments are mainly performed in the red boxed area with the sub-picosecond pulse durations.

**Table S1** Theoretical estimation for electron numbers in the each electron pocket for getting the subpicosecond time-resolution in UTEM at IOP, these calculations are based on the method reported ref.2 and the relevant UTEM parameters used in our experiments.

| Cathode Diameter | Operation voltage/Electron Number per Pulse | | |
| --- | --- | --- | --- |
|  | 120kV | 160kV | 200kV |
| 16μm | 2.56 | 3.84 | 5.12 |
| 50μm | 25 | 37.5 | 50 |
| 100μm | 100 | **150** | 200 |

**4. Micrograph for carbon nano-particles obtained in the UTEM mode**


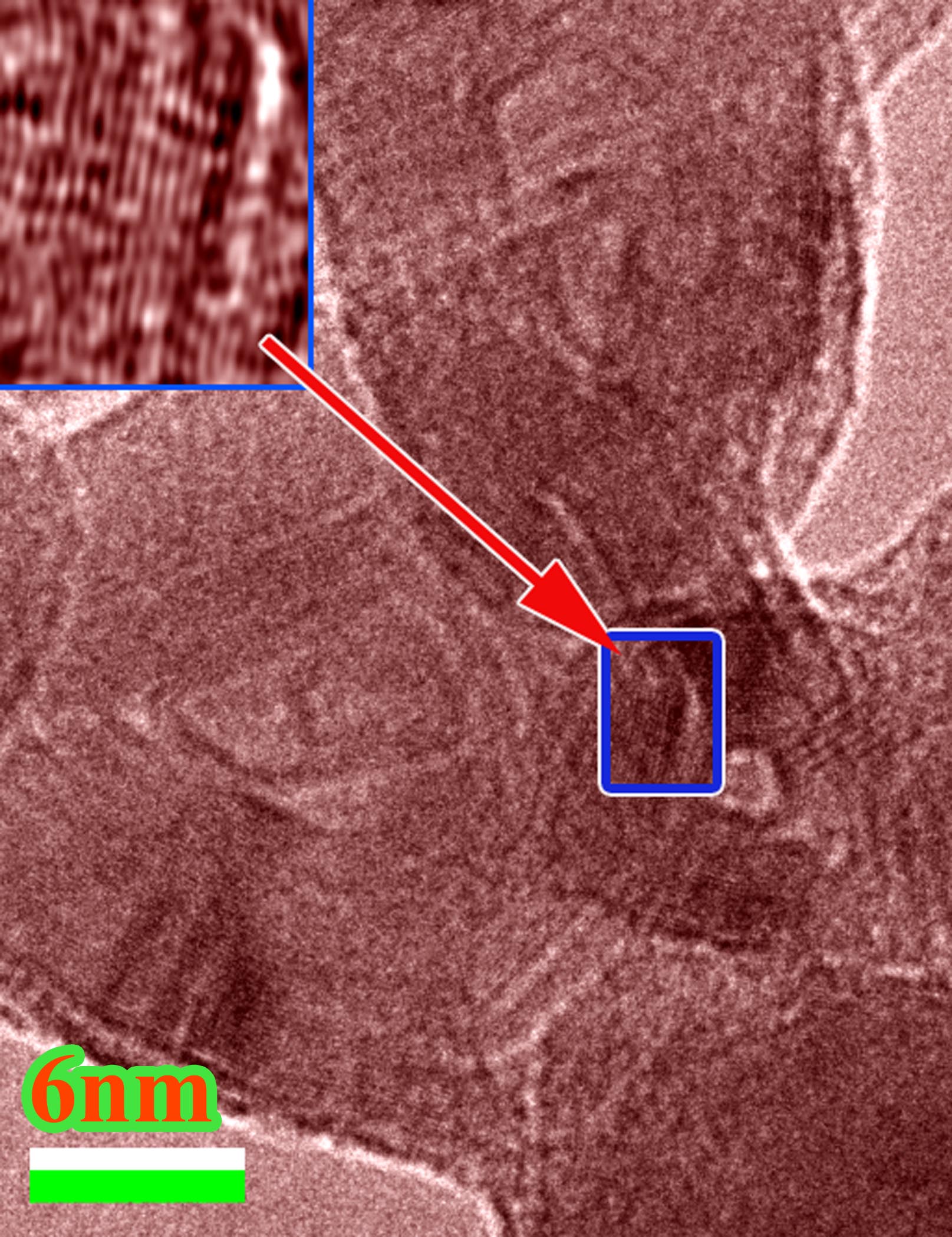


**Figure S3** **Micrograph for carbon nano-particles obtained in the UTEM mode**. The lattice fringe d = 0.34nm can be observed at the center area (Cathode laser: Wave length λ=355nm, repetition rate: 80MHz).

**References**

1. Dwyer, J. R. *et al*. Femtosecond electron diffraction: 'making the molecular movie'. *Phil. Trans. R. Soc. A* **364,** 741-778 (2006).

2. King, W. E. *et al*. Ultrafast electron microscopy in materials science, biology, and chemistry. *J. Appl. Phys.* **97,** 111101(2005).
